# 2024 Public Health Actions to Reduce the Burden of Asthma: Influenza and COVID-19 Vaccination Uptake Among People with Asthma

**DOI:** 10.5888/pcd21.240058

**Published:** 2024-08-08

**Authors:** Hannah Jaffee, Sanaz Eftekhari, Melanie Carver

**Affiliations:** 1 The Asthma and Allergy Foundation of America, Arlington, Virginia

## Abstract

This study sought to identify COVID-19 and influenza vaccination rates and barriers among people with asthma. The Asthma and Allergy Foundation of America (AAFA) conducted an online survey from April to May in 2022 among a convenience sample of 350 individuals with asthma. Most survey respondents reported that they had received an influenza vaccine for the 2021–2022 flu season (77%) and at least 1 dose of a COVID-19 vaccine (87%). Age, gender, race and ethnicity, and household income were significantly associated with influenza vaccination. Age and urban–rural classification were associated with COVID-19 vaccination. Access issues were not commonly reported as vaccination barriers, highlighting educational opportunities.

**Summary**

**What is already known on this topic?**

Optimal asthma management, including vaccination, can help people with asthma during respiratory virus seasons to protect against infection and severe symptoms.

**What is added by this report?**

The study highlights significant differences in vaccination rates for people with asthma across demographic categories. Access challenges were not commonly reported as reasons for not getting vaccinated.

**What are the implications for public health practice?**

Findings identify differences in influenza and COVID-19 vaccination rates based on demographic factors. The results of this study can inform the development and implementation of tailored educational and communication efforts to improve vaccination rates in these populations.

## Objective

The onset of the COVID-19 pandemic in March 2020 resulted in major disruption to everyday life. Additionally, the threat of a “tripledemic” — marked by a high number of cases of COVID-19, influenza, and respiratory syncytial virus (RSV) — continued into 2023 (1). Previous literature shows that respiratory infections can be more serious for individuals with asthma, as infection can exacerbate asthma symptoms and lead to poorer health outcomes (2,3). Therefore, practicing optimal asthma management during respiratory virus seasons can be beneficial for people with asthma (4). Vaccines have been shown to help protect people with asthma against respiratory infections and lessen symptom severity if an infection occurs (5). However, previous literature from other countries suggests that influenza and COVID-19 vaccination rates in adults with asthma is suboptimal (6,7). Although national vaccine surveillance data are widely available (8), little is known about influenza and COVID-19 vaccination uptake among people with asthma in the US. We sought to gauge vaccination rates among people with asthma in the US and understand what, if any, demographic differences exist in vaccination rates and barriers in this population.

## Methods

The Asthma and Allergy Foundation of America (AAFA), a patient advocacy organization, conducted an online survey from April 6 to May 31, 2022, to assess influenza and COVID-19 vaccination behaviors and barriers among people with asthma and allergies. A convenience sample of people with self-reported diagnoses of asthma and allergies, as well as caregivers (eg, parents, guardians) of people diagnosed with these conditions, was surveyed for participation. Participants were recruited through AAFA’s e-newsletters and social media posts. To qualify for the survey, participants needed to live in the US and be a legal adult in their state of residence. Participants also needed to be a person with, or a caregiver to a person with, a self-reported diagnosis of asthma or allergies. Respondents were screened for eligibility through self-reported responses. The research protocol was reviewed and determined exempt by Advarra Institutional Review Board.

This analysis focused on adults with asthma because of an increased risk of poor asthma outcomes from respiratory infection. Participants responded on their own behalf. Data on adults with asthma were identified for analysis based on self-reported diagnosed conditions. Outcome variables included vaccination status for the 2020–2021 and 2021–2022 influenza seasons, and initial and subsequent COVID-19 vaccinations. To assess vaccination barriers, unvaccinated respondents selected barriers from a list which were then categorized into perceptual (eg, beliefs about vaccine safety or efficacy) and technical (eg, access, scheduling issues) categories. Descriptive statistics on vaccination rates and barriers were analyzed by using SPSS version 29.0 (IBM). Chi-square tests of independence and Fisher exact tests were used to examine relationships between vaccination rates and barriers and self-reported age, gender, race and ethnicity, annual household income, and urban–rural classification. Statistical significance was set at *P* ≤ .05 for Pearson χ^2^ and Fisher exact tests to identify relationships between vaccination rates and demographic factors.

## Results

Of the 1,664 people who began the survey, 537 completed the survey for a completion rate of 32%. Among completed respondents, 350 were adults living with asthma, predominantly identifying as White, women, suburban residents, and having an annual household income exceeding $50,000 (Table 1).

**Table 1:** Demographic Characteristics of Survey Respondents, by Asthma Status, Online Survey of the Asthma and Allergy Foundation of America, April 6 to May 31, 2022

Characteristic	Total (N = 537)	Asthma (n = 350)	No Asthma (n = 187)
	No. (%)		
**Age, y**			
≤25	11 (2)	7 (2)	4 (2)
26–41	126 (23)	66 (19)	60 (32)
42–57	226 (42)	133 (38)	93 (50)
58–76	162 (30)	135 (39)	27 (14)
≥77	12 (2)	9 (3)	3 (2)
**Gender**			
Man	47 (9)	39 (11)	8 (4)
Woman	478 (89)	303 (87)	175 (94)
Nonbinary or gender nonconforming	2 (0)	1 (0)	1 (1)
Prefer not to answer	10 (2)	7 (2)	3 (2)
**Race and ethnicity**			
Indigenous American, American Indian, or Alaska Native	12 (2)	11 (3)	1 (<1)
Asian	14 (3)	7 (2)	7 (4)
Black or African American	28 (5)	21 (6)	7 (4)
Hispanic or Latino/a	37 (7)	23 (7)	14 (7)
Middle Eastern or North African	4 (1)	2 (<1)	2 (1)
Native Hawaiian or Pacific Islander	1 (<1)	1 (<1)	0
White	404 (75)	262 (75)	142 (76)
Other	8 (1)	5 (1)	3 (2)
Prefer not to answer	29 (5)	18 (5)	11 (6)
**Annual household income, $**			
<50,000	76 (14)	61 (17)	15 (8)
50,000–99,999	152 (28)	113 (32)	39 (21)
≥100,000	194 (36)	101 (29)	93 (50)
Prefer not to answer	115 (21)	75 (21)	40 (21)
**Urban–rural classification**			
Urban	101 (19)	79 (23)	22 (12)
Rural	117 (22)	70 (20)	47 (25)
Suburban	302 (56)	188 (54)	114 (61)
Prefer not to answer	17 (3)	13 (4)	4 (2)

More than three-quarters of respondents with asthma received an influenza vaccine for the 2020–2021 (78%) and the 2021–2022 (77%) influenza seasons. Vaccination rates for the 2020–2021 influenza season were higher among respondents aged 58 to 76 years than among those aged 26 to 57 years (*P* < .001). For the 2021–2022 influenza season, vaccination rates were higher among respondents aged 58 to 76 years than among those aged 26 to 57 years (*P* < .001), among men than among women (*P* = .05), among White respondents than among Hispanic or Latino/a respondents (*P* = .04), and among respondents with an annual household income of $100,000 or more than among those with an annual household income less than $50,000 (*P* = .01) (Table 2).

**Table 2:** Influenza and COVID-19 Vaccination Rates Among People with Asthma, by Respondent Characteristics, Online Survey of the Asthma and Allergy Foundation of America, April 6 to May 31, 2022^a^

Characteristic	No.	Received an influenza vaccination for the October 2020–May 2021 influenza season, n (%)	Received an influenza vaccination for the October 2021–May 2022 influenza season, n (%)	Received ≥1 dose of a COVID-19 vaccine, n (%)	Fully vaccinated for COVID-19, n (%)^b^	Fully vaccinated and received a booster dose for COVID-19, n (%)
**Total^c^**	350	272 (78)	269 (77)	304 (87)	299 (85)	257 (73)
**Age, y**						
≤25	7	6 (86)	7 (100)	7 (100)	7 (100)	7 (100)
26–41	66	40 (61)	40 (61)	51 (77)	48 (73)	40 (61)
42–57	133	96 (72)	94 (71)	108 (81)	107 (80)	87 (65)
58–76	135	121 (90)	119 (88)	130 (96)	129 (96)	115 (85)
≥77	9	9 (100)	9 (100)	8 (89)	8 (89)	8 (89)
*P* value	—	<.001^d^	<.001^d^	<.001^d^	<.001^d^	.30^d^
**Gender**						
Man	39	34 (87)	35 (90)	34 (87)	34 (87)	32 (82)
Woman	303	235 (78)	230 (76)	266 (88)	261 (86)	222 (73)
*P* value	—	.22	.05	>.99^d^	.60	.19^d^
**Race and ethnicity**						
Indigenous American, American Indian, or Alaska Native	11	9 (82)	7 (64)	10 (91)	10 (91)	8 (73)
Asian	7	4 (57)	4 (57)	5 (71)	5 (71)	5 (71)
Black or African American	21	15 (71)	16 (76)	19 (91)	19 (91)	17 (81)
Hispanic or Latino/a	23	14 (61)	13 (57)	18 (78)	18 (78)	15 (65)
White	262	211 (81)	212 (81)	234 (89)	230 (88)	202 (77)
Other^e^	8	5 (63)	6 (75)	6 (75)	6 (75)	5 (63)
*P* value	—	.09^d^	.04^d^	.15^d^	.27^d^	.80^d^
**Annual household income, $**						
<50,000	61	43 (70)	40 (66)	48 (79)	48 (79)	37 (61)
50,000–99,999	113	93 (82)	89 (79)	104 (92)	102 (90)	84 (74)
≥100,000	101	84 (83)	86 (85)	89 (88)	89 (88)	86 (85)
*P* value	—	.08	.01	.12	.24	.001
**Urban–rural classification**						
Urban	79	65 (82)	63 (80)	74 (94)	72 (91)	66 (84)
Rural	70	50 (71)	48 (69)	53 (76)	53 (76)	39 (56)
Suburban	188	148 (79)	151 (80)	169 (90)	167 (89)	147 (78)
*P* value	—	.20	.12	.003	.01	.009

Abbreviation: — , not applicable.

a *P* values based on χ^2^ test of independence and Fisher exact test; significance set at *P* ≤ .05.

b “Fully vaccinated” was defined as having completed a primary series of COVID-19 vaccinations.

c Respondent characteristics may not add up to total due to exclusion of “prefer not to answer” categories from analysis, as well as categories in which n < 5.

d Fisher exact test was used because ≥20% of expected cell values were n < 5.

e Includes Middle Eastern or North African, Native Hawaiian or Pacific Islander, and other.

Most respondents with asthma reported receiving 1 or more doses of a COVID-19 vaccine (87%), completing a primary series for COVID-19 (85%), and completing a primary series for COVID-19 with a booster dose (73%). Initial COVID-19 vaccination rates were higher for respondents aged 58 to 76 years compared with those aged 26 to 57 years (*P* < .001) and for respondents in urban and suburban areas compared with those in rural areas (*P* = .003). The same differences were seen for full COVID-19 vaccination in age (*P* < .001) and urban–rural classification (*P* = .01). COVID-19 booster rates were higher for respondents with an annual household income of $100,000 or more compared with those with an annual household income under $50,000 (*P* = .001) and for respondents in urban and suburban areas compared with those in rural areas (*P* = .009) (Table 2).

Among respondents with asthma who did not receive an influenza or COVID-19 vaccine, no significant demographic differences were found in citing perceptual or technical barriers. Technical barriers were less commonly selected as barriers for influenza vaccines and were not selected by any respondents as barriers for COVID-19 vaccines (Table 3).

**Table 3:** Reasons for Not Receiving an Influenza or COVID-19 Vaccination Among Unvaccinated Respondents, Online Survey of the Asthma and Allergy Foundation of America, April 6 to May 31, 2022^a^

Characteristic	Unvaccinated for 2021–2022 Influenza (N = 81)^b^	Reason for Not Receiving an Influenza Vaccine, Perceptual, n (%)^c^	Reason for Not Receiving an Influenza Vaccine, Technical, n (%)^c^	Unvaccinated for COVID-19 (N = 46)^b^	Reason for Not Receiving a COVID-19 Vaccine, Perceptual, n (%)^d^	Reason for Not Receiving a COVID-19 Vaccine, Technical, n (%)^d^
**Age, y**						
≤25	0	0	0	0	0	0
26–41	26	22 (85)	4 (15)	15	15 (100)	0
42–57	39	31 (79)	8 (21)	24	24 (100)	0
58–76	16	13 (81)	3 (19)	4	4 (100)	0
≥77	0	0	0	1	1 (100)	0
*P* value	—	<.93^e^		—	—	
**Gender**						
Man	4	4 (100)	0	4	4 (100)	0
Woman	73	59 (81)	14 (19)	36	36 (100)	0
*P* value	—	>.99^e^		—	—	
**Race and ethnicity**						
Indigenous American, American Indian, or Alaska Native	4	4 (100)	0	1	1 (100)	0
Asian	3	3 (100)	0	2	2 (100)	0
Black or African American	5	5 (100)	0	2	2 (100)	0
Hispanic or Latino/a	10	7 (70)	3 (30)	5	5 (100)	0
White	50	42 (84)	8 (16)	216	216 (100)	0
Other^f^	2	1 (50)	1 (50)	2	2 (100)	0
*P* value	—	.41^e^		—	—	
**Annual household income, $**						
<50,000	21	18 (86)	3 (14)	11	11 (100)	0
50,000–99,999	24	19 (79)	5 (21)	9	9 (100)	0
≥100,000	15	13 (87)	2 (13)	12	12 (100)	0
*P* value	—	.83^e^		—	—	
**Urban–rural classification**						
Urban	16	12 (75)	4 (25)	5	5 (100)	0
Rural	22	21 (95)	1 (5)	16	16 (100)	0
Suburban	37	29 (78)	8 (22)	18	18 (100)	0
*P* value	—	.17^e^		—	—	

Abbreviation: — , not applicable.

a *P* values based on χ^2^ test of independence and Fisher exact test; significance set at *P* ≤ .05.

b Totals for respondent characteristics may not add up to overall total due to exclusion of “prefer not to answer” categories from analysis, as well as categories in which n < 5.

c Responses were categorized as technical if respondent selected “I do not have easy access to an influenza shot clinic” or “I haven’t found the time to schedule an appointment.” All other responses were categorized as perceptual.

d Responses were categorized as technical if respondent selected “I have scheduled an appointment for the vaccine for a future date,” “I have had trouble finding appointment(s) to get a vaccine,” “I have trouble navigating the process to sign up for a vaccine,” or “It is difficult for me to travel to a vaccination site.” All other responses were categorized as perceptual.

e Fisher exact test was used because ≥20% of expected cell values were n < 5.

f Includes Middle Eastern or North African, Native Hawaiian or Pacific Islander, and other.

## Discussion

We investigated influenza and COVID-19 vaccination rates among a subgroup of people with asthma, and although influenza and COVID-19 vaccination rates among this group exceeded national averages (8), we found significant demographic differences. Respondents aged 58 to 76 years were more likely to be vaccinated for influenza and COVID-19 compared with younger respondents, and respondents in urban and suburban areas were more likely to be vaccinated for COVID-19 compared with those in rural areas. These demographic differences mirror national demographic differences in vaccination rates (8). Reasons for variation may include earlier COVID-19 vaccine eligibility for older adults and better access to vaccine resources in urban and suburban communities.

We also examined barriers to vaccination among unvaccinated respondents with asthma. Perceptual barriers (eg, beliefs about vaccine safety or efficacy) outweighed technical barriers (eg, access, scheduling issues), aligning with findings from a previous study among Canadian adults with asthma (9). These results indicate opportunities for education on vaccine safety and efficacy, particularly for people with asthma.

Our study has limitations. We relied on a convenience sample that may be more likely to be vaccinated than the total population of people with asthma. Additionally, most survey respondents were higher-income, White women and therefore not representative of the national population of people with asthma, which is more diverse in income, race and ethnicity, and gender (10). Statistical testing was limited by sample size variations across demographic groups, potentially obscuring significant differences that may be seen in a more diverse sample. Lastly, the survey relied on self-reported data, which is prone to several biases including social desirability and recall bias.

Despite these limitations, the study contributes valuable insights into vaccination behaviors among people with asthma, a group susceptible to severe illness from respiratory infections. It represents the first attempt, to the authors’ knowledge, to analyze influenza and COVID-19 vaccination behaviors in this population in the US. Future research can aim for nationally representative samples to better understand demographic differences in this population, as generational and cultural beliefs can further influence vaccination behavior (11,12). Additionally, future research can examine differences in vaccination rates between people with and without asthma to understand differences in these populations.

Our study offers insights into vaccination behaviors of a subgroup of people with asthma to inform future research. The findings also highlight opportunities for improved vaccine communication strategies to reduce prevalence and severe outcomes of respiratory diseases across demographic groups among people with asthma.
